# Digital PCR-based evaluation of nucleic acid extraction kit performance for the co-purification of cell-free DNA and RNA

**DOI:** 10.1186/s40246-022-00446-4

**Published:** 2022-12-31

**Authors:** Jill Deleu, Kathleen Schoofs, Anneleen Decock, Kimberly Verniers, Sofie Roelandt, Angie Denolf, Joke Verreth, Bram De Wilde, Tom Van Maerken, Katleen De Preter, Jo Vandesompele

**Affiliations:** 1 OncoRNALab, Cancer Research Institute Ghent (CRIG), Ghent, Belgium; 2 Department of Biomolecular Medicine, Ghent University, Ghent, Belgium; 3 Translational Oncogenomics and Bioinformatics Lab, Cancer Research Institute Ghent (CRIG), Ghent, Belgium; 4 Center for Medical Biotechnology, VIB-UGent, Ghent, Belgium; 5 Department of Paediatric Haematology Oncology and Stem Cell Transplantation, Ghent University Hospital, Ghent, Belgium; 6 Department of Laboratory Medicine, AZ Groeninge, Kortrijk, Belgium

**Keywords:** Liquid biopsies, Co-purification, Cell-free RNA, Extracellular RNA, Cell-free DNA, Mutation detection, Digital PCR, Plasma, Cancer

## Abstract

**Background:**

Blood plasma, one of the most studied liquid biopsies, contains various molecules that have biomarker potential for cancer detection, including cell-free DNA (cfDNA) and cell-free RNA (cfRNA). As the vast majority of cell-free nucleic acids in circulation are non-cancerous, a laboratory workflow with a high detection sensitivity of tumor-derived nucleic acids is a prerequisite for precision oncology. One way to meet this requirement is by the combined analysis of cfDNA and cfRNA from the same liquid biopsy sample. So far, no study has systematically compared the performance of cfDNA and cfRNA co-purification to increase sensitivity.

**Results:**

First, we set up a framework using digital PCR (dPCR) technology to quantify cfDNA and cfRNA from human blood plasma in order to compare cfDNA/cfRNA co-purification kit performance. To that end, we optimized two dPCR duplex assays, designed to quantify both cfDNA and cfRNA with the same assays, by ensuring that primers and probes are located within a highly abundant exon. Next, we applied our optimized workflow to evaluate the co-purification performance of two manual and two semi-automated methods over a range of plasma input volumes (0.06–4 mL). Some kits result in higher nucleic acid concentrations in the eluate, while consuming only half of the plasma volume. The combined nucleic acid quantification systematically results in higher nucleic acid concentrations as compared to a parallel quantification of cfDNA and cfRNA in the eluate.

**Conclusions:**

We provide a framework to evaluate the performance of cfDNA/cfRNA co-purification kits and have tested two manual and two semi-automated co-purification kits in function of the available plasma input amount and the intended use of the nucleic acid eluate. We demonstrate that the combined quantification of cfDNA and cfRNA has a benefit compared to separate quantification. We foresee that the results of this study are instrumental for clinical applications to help increase mutation detection sensitivity, allowing improved disease detection and monitoring.

**Supplementary Information:**

The online version contains supplementary material available at 10.1186/s40246-022-00446-4.

## Background

For an increasing number of malignancies, the mutation status of particular genes is crucial for diagnosis or treatment decision [1]. Assessing the mutation status of a tumor typically requires a tissue biopsy, which comes with discomfort and risk for the patient. Moreover, a tissue biopsy does not capture genetic heterogeneity well and is not compatible with longitudinal profiling. For these reasons, liquid biopsies are heralded as a promising alternative for both the patient and clinician [2–4].

Blood plasma is one of the most studied liquid biopsies as tumor-derived molecules end up in circulation by either active or passive release from tumor cells [5]. However, also healthy cells release their content into the bloodstream and as such, only a small fraction of circulating molecules is originating from the tumor. Moreover, for patients with small or slow-growing tumors, only a limited amount of nucleic acids may end up in the bloodstream, leading to reduced analytical sensitivity [6]. Consequently, a laboratory workflow for liquid biopsies that comes with high mutation detection sensitivity is a prerequisite for precision oncology. Several strategies exist to enhance the analytical sensitivity, ranging from higher blood plasma input volumes for extraction to advanced error-correcting molecular methods [7]. Recently, the combined analysis of cell-free DNA and RNA has been proposed as another means of increasing mutation detection sensitivity [8–11]. However, most studies have focused on RNA originating only from extracellular vesicles (EVs), thereby ignoring a significant part of the cell-free RNA (cfRNA) repertoire outside vesicles. Although EV concentration is thought to be increased in cancer patients’ plasma, purification practices are not standardized among laboratories, are time consuming, and result in loss of material when trying to obtain highly pure and well-characterized EVs [12, 13]. To increase mutation detection sensitivity, it may be advantageous to co-purify cell-free DNA (cfDNA) and cfRNA from the same aliquot of neat plasma. Even if separate downstream analyses for DNA and RNA are desired, co-purification is advantageous, because it is cost and time effective, and allows maximal use of valuable patient samples.

**Fig. 1:**
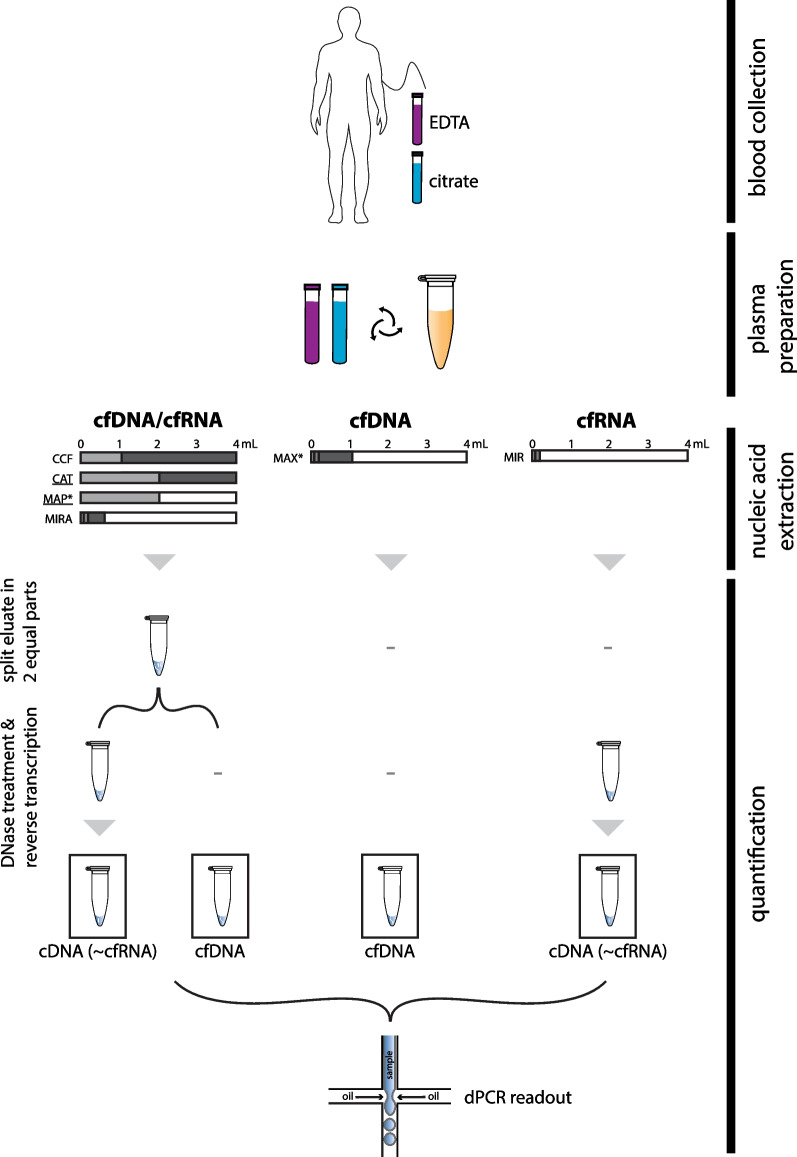
Experimental design to evaluate (co-)purification kits. Blood from healthy donors, collected in EDTA or citrate tubes, was processed into plasma using a 2-spin protocol. Different plasma volumes were used as input for the different kits: 1 mL and 4 mL for CCF; 2 mL and 4 mL for CAT; 2 mL for MAPss and MAPds; 0.06 mL, 0.2 mL and 0.6 mL for MIRA; 0.06 mL, 0.2 mL and 1 mL for MAX; 0.06 mL and 0.2 mL for MIR. One half of the eluate was DNase treated and reverse transcribed for cfRNA quantification, while the second half remained untouched for cfDNA quantification. Quantification of nucleic acids was performed by digital PCR. *MAP and MAX eluates were concentrated using Vivacon columns and vacuum centrifugation, respectively. Underlined kits (CAT and MAP) are semi-automated procedure systems. *CCF*: QIAamp ccfDNA/RNA Kit, *CAT*: iCatcher Circulating cfDNA/cfRNA 4000 kit, *MAP*: MagNA Pure 24 Total NA Isolation Kit, *MIRA*: miRNeasy Serum/Plasma Advanced Kit, *MAX*: Maxwell ccfDNA LV Plasma Kit, *MIR*: miRNeasy Serum/Plasma Kit

So far, no study has systematically compared the performance of cfDNA and cfRNA co-purification technologies. The purpose of the present study is to provide a framework to compare different purification methods using digital PCR (dPCR) technology. Using healthy donor plasma, we compared two manual and two semi-automated co-purification methods and provide an evaluation in function of the available plasma input amount and the intended use of the nucleic acid eluate (Fig. 1).

## Results

### A framework for dPCR-based assessment of cfDNA/cfRNA co-purification kit performance

**Fig. 2:**
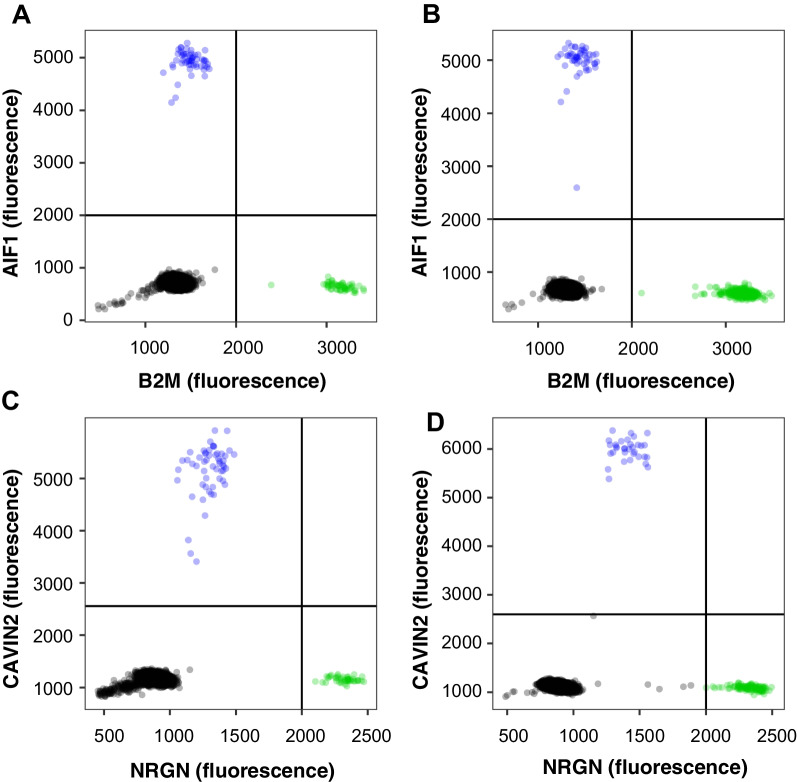
Representative two-color plots of two dPCR duplex assays for quantification of cfDNA (**A**, **C**) and cfRNA (**B**, **D**) shows a nice distribution of positive and negative partitions. Data from nucleic acids co-purified with MIRA0.6 from healthy donor plasma (blood collected in EDTA tubes). **A**, **B** Duplex assay for AIF1 (FAM, blue) and B2M (HEX, green). **C**, **D** Duplex assay for CAVIN2 (FAM, blue) and NRGN (HEX, green). Green and blue dots represent positive partitions of the two genes, respectively. Black dots represent negative partitions

To compare the plasma cfDNA/cfRNA co-purification performance of manual and semi-automated procedures, a dPCR-based method was introduced for accurate and precise determination of the cfDNA and cfRNA concentration using two duplex assays targeting four human genes: CAVIN2 (HEX) / NRGN (FAM) and AIF1 (FAM) / B2M (HEX) (see Methods). Both duplex assays show positive partitions that are well separated from the negative partitions for cfDNA and cfRNA isolated from healthy human donor plasma. A representative example is shown in Fig. 2 for cfDNA/cfRNA co-purified from 0.6 mL of EDTA blood plasma from one donor using the miRNeasy Serum/Plasma Advanced Kit (MIRA0.6).

The framework, as represented in Fig. 1, outlines the steps to compare different co-purification kits on the basis of dPCR quantification. To allow for reliable cfRNA quantification, all DNA should be removed from the sample. Therefore, DNase treatment efficacy was evaluated on the eluates from each kit (Additional file 1: Fig. S1).

In the following sections, we will first discuss the co-purification performance of the evaluated kits, followed by ways to improve detection sensitivity for cfDNA and cfRNA quantification, and a demonstration of the added value of a combined cfDNA/cfRNA quantification instead of a parallel quantification of cfDNA and cfRNA.

### Assessment of cfDNA/cfRNA co-purification performance of four commercial kits

The optimized workflow was used to assess the performance of six different commercially available (co-)purification kits: (1) MIRA, (2) QIAamp ccfDNA/RNA Kit (CCF), (3) iCatcher Circulating cfDNA/cfRNA 4000 kit (CAT), (4) MagNA Pure 24 Total NA Isolation Kit with the cfNA ss 2000 and cfNA ds 2000 purification protocols (MAPss and MAPds, respectively), (5) Maxwell ccfDNA LV Plasma Kit (MAX) and (6) miRNeasy Serum/Plasma Kit (MIR). The performance of the four co-purification kits (CAT, MIRA, MAP, CCF) was compared with kits developed to extract only cfDNA (MAX) or cfRNA (MIR). For each kit, a range of plasma input volumes were used to assess kit performance (Fig. 1, Additional file 2: Table S1).

**Fig. 3:**
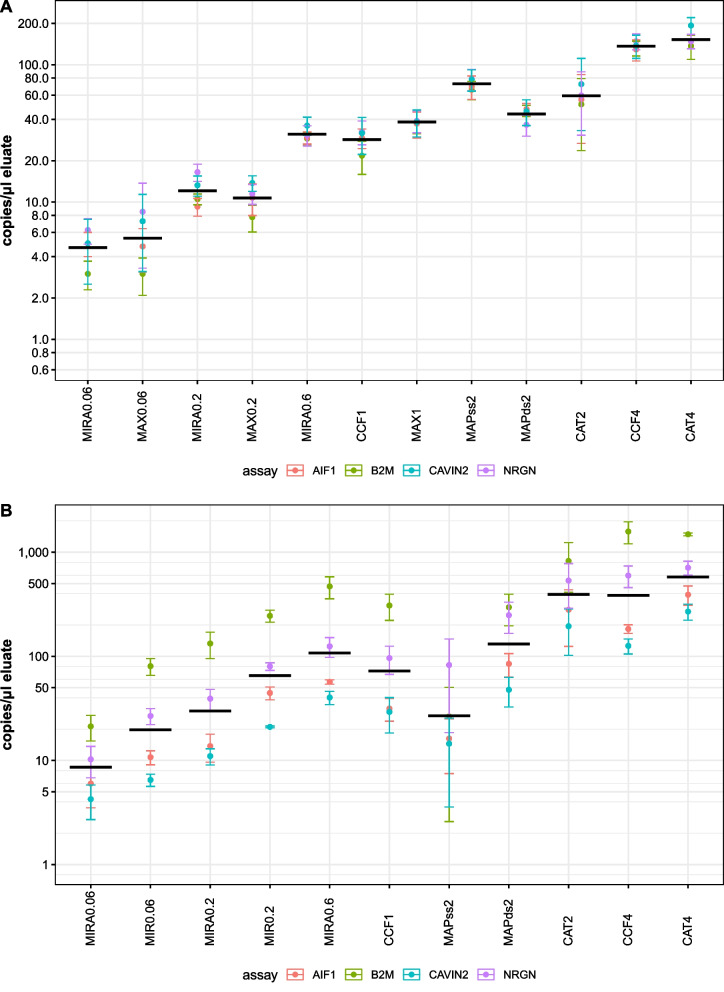
Quantification of cfDNA (**A**) and cfRNA (**B**) extracted from healthy donor plasma with six (co-)purification kits using different input volumes. Per assay the mean concentration of the tube and donors was taken, error bars indicate standard errors. Black horizontal lines indicate geometric means of the four assays. *MIR*: miRNeasy Serum/Plasma kit, *MIRA*: miRNeasy Serum/Plasma Advanced kit, *MAX*: Maxwell ccfDNA plasma kit, *CCF*: QIAamp ccfDNA/RNA Kit, *MAPss*: MagNA Pure 24 Total NA Isolation Kit (Single Strand protocol), *MAPds*: MagNA Pure 24 Total NA Isolation Kit (Double Strand protocol), *CAT*: iCatcher Circulating cfDNA/cfRNA. Numbers after kit abbreviation indicate plasma input volumes in mL

The cfDNA and cfRNA concentration (copies per µl eluate) was determined by dPCR (Fig. 3). In general, results show little variation between donors and blood collection tube types, as can be seen by the small error bars (showing the standard deviation of the geometric mean of all donors and blood collection tubes). As expected, we observe constant cfDNA concentrations as assessed by the four assays, while cfRNA concentrations are widely different among assays, reflecting the dynamic nature of cfRNA. Also, the concentration of extracted nucleic acids in the eluate increases proportionally with higher input volumes when using the same kit (Fig. 3, Additional file 3: Table S2).

Furthermore, we compared the co-purification performance of the evaluated kits by measuring the cfDNA and cfRNA concentration and determining the cfDNA and cfRNA yield (concentration multiplied by the eluate volume) (Additional file 4: Table S3). All co-purification kits were capable of co-purifying cfDNA and cfRNA. While most of the co-purification kits resulted in reproducible eluate volumes (MIRA, CCF and MAP), eluate volumes after purification with CAT were ranging between 18 and 27 µl. In Additional file 4: Table S3, concentration and yield for each kit and input volume are calculated, followed by rescaling to the maximum concentration or yield (CAT4 in all cases) to obtain relative values (percentages). For cfRNA, the highest concentrations and yields are obtained with CAT4, CAT2 and CCF4 kit. For cfDNA, the highest concentrations and yields are obtained with CAT4, CCF4 and MAPss2 kit. Additional kit characteristics and remarks are provided in Additional file 2: Table S1.

### Assessing cfDNA size distribution using microfluid electrophoresis

**Fig. 4:**
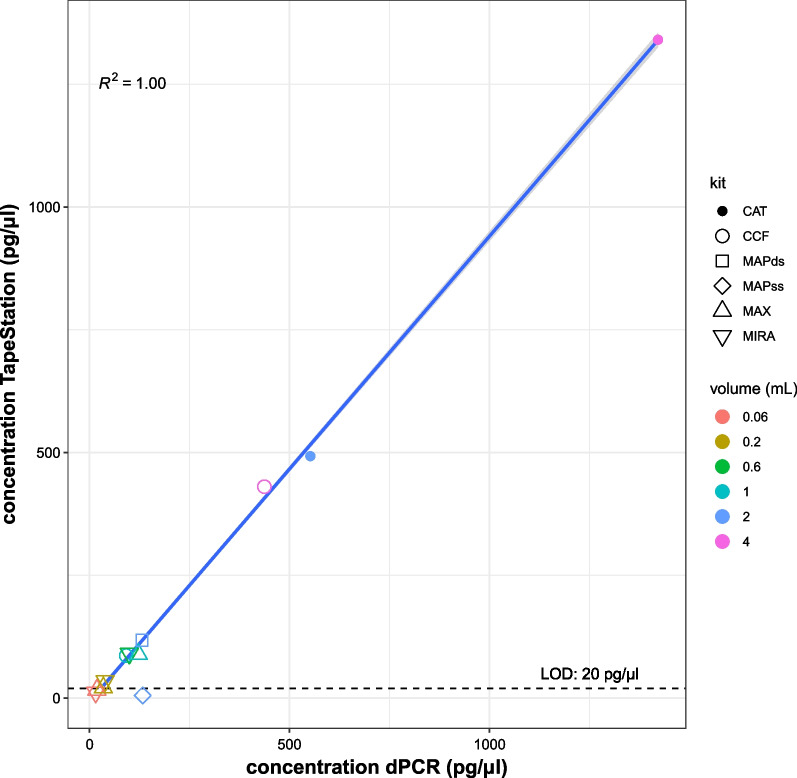
cfDNA concentration measured with microfluid electrophoresis (*y*-axis) and dPCR (*x*-axis) show a good correlation. Each dot represents the mean of the donors and blood tubes. Concentration dPCR was calculated by taking the mean of the four assays in the input volume used for dPCR. Concentrations from QuantaSoft software (copies/µl) were converted to pg/µl by multiplying by 3.2 pg. Sample means below LOD of TapeStation were excluded for calculating *R*^2^ (3/12)

To ascertain that the measured DNA is cell-free in origin and not high-molecular cellular DNA, fragment size analysis using microfluid electrophoresis (TapeStation) was performed. cfDNA has a typical average fragment length of ~ 170 bp, whereas larger fragments (> 700 bp) originate from lysed cells and are defined as high molecular weight (HMW) DNA. For 30/46 samples with cfDNA concentration above the limit of detection for TapeStation (20 pg/µl), the cfDNA percentage is between 64 and 94%, indicating overall good quality (pure) cfDNA and low fractions of HMW DNA (Additional file 5: Fig. S2). Furthermore, cfDNA concentrations as determined above LOD by TapeStation correlate well with cfDNA concentrations measured by dPCR (Fig. 4).

### Increasing detection sensitivity by increasing cfDNA/cfRNA template volume in a digital PCR reaction

**Fig. 5:**
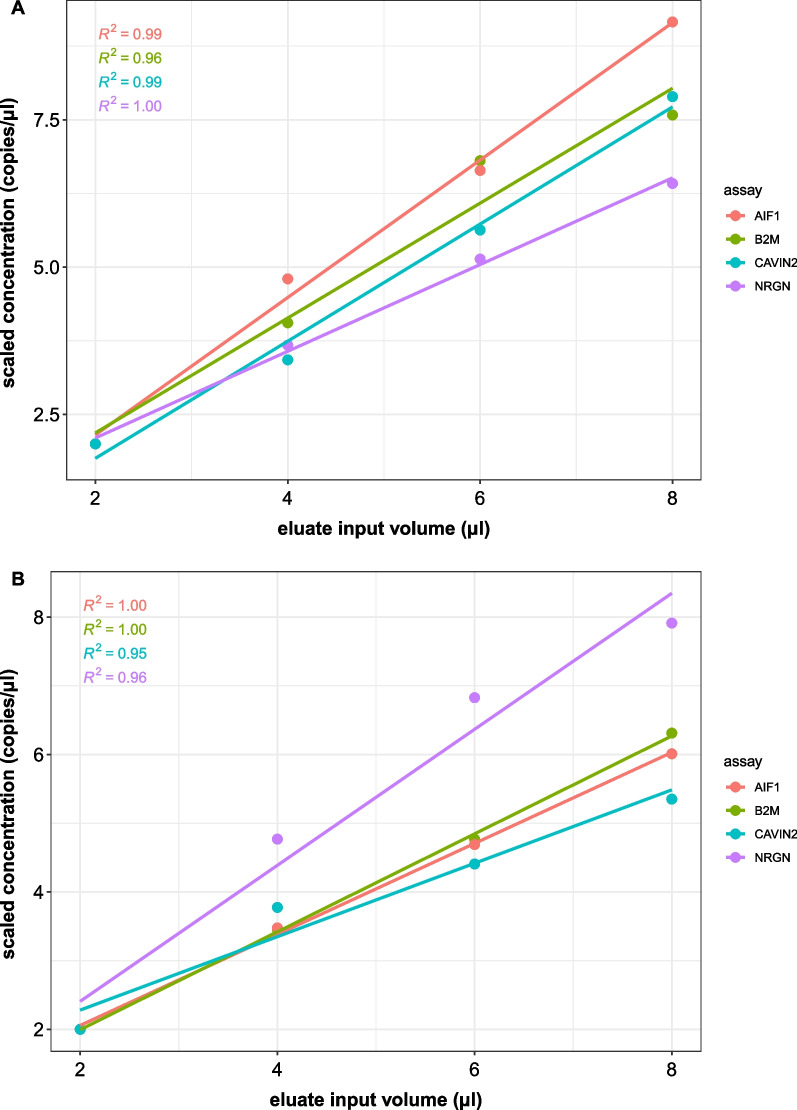
Increasing the fraction of eluate in the dPCR reaction volume does not inhibit quantification. cfDNA (**A**) and cDNA (**B**) of healthy donor template was added at different percentages of the total reaction volume. For each condition the mean concentration of duplicates is shown

Blood plasma is characterized by low cfDNA and cfRNA concentration. To increase the detection sensitivity with dPCR, the template input volume in a 20 µl reaction can be increased. A more concentrated mix of primers and probes can be made and added to the reaction mix as a small volume, enabling the addition of a higher cfDNA/cfRNA eluate volume. However, increasing the eluate input volume may cause inhibition of the dPCR reaction, which may be assay dependent [14]. Therefore, the inhibitory effect of the eluate (cfDNA/cfRNA template molecules with possible carry-over products from sample matrix) in a dPCR reaction was evaluated for the four optimized dPCR assays with input volumes taking up 10–40% of the total reaction volume. The assays used in this study did not show inhibition for cfDNA (Fig. 5A) nor cDNA (~ cfRNA) (Fig. 5B) when increasing eluate input volumes. cfDNA and cfRNA used for this experiment was co-purified using the MIRA kit with 0.6 mL plasma as input volume. As a proof-of-principle, these results show it is possible to confidently maximize input volume up to 40% of total reaction volume using these four assays (for MIRA eluates using the 2 × ddPCR Supermix for Probes and QX100 dPCR instrument). We recommend that users evaluate eluate input inhibition for their own assays and dPCR reaction conditions.

### Combined quantification of cfDNA and cfRNA

**Fig. 6:**
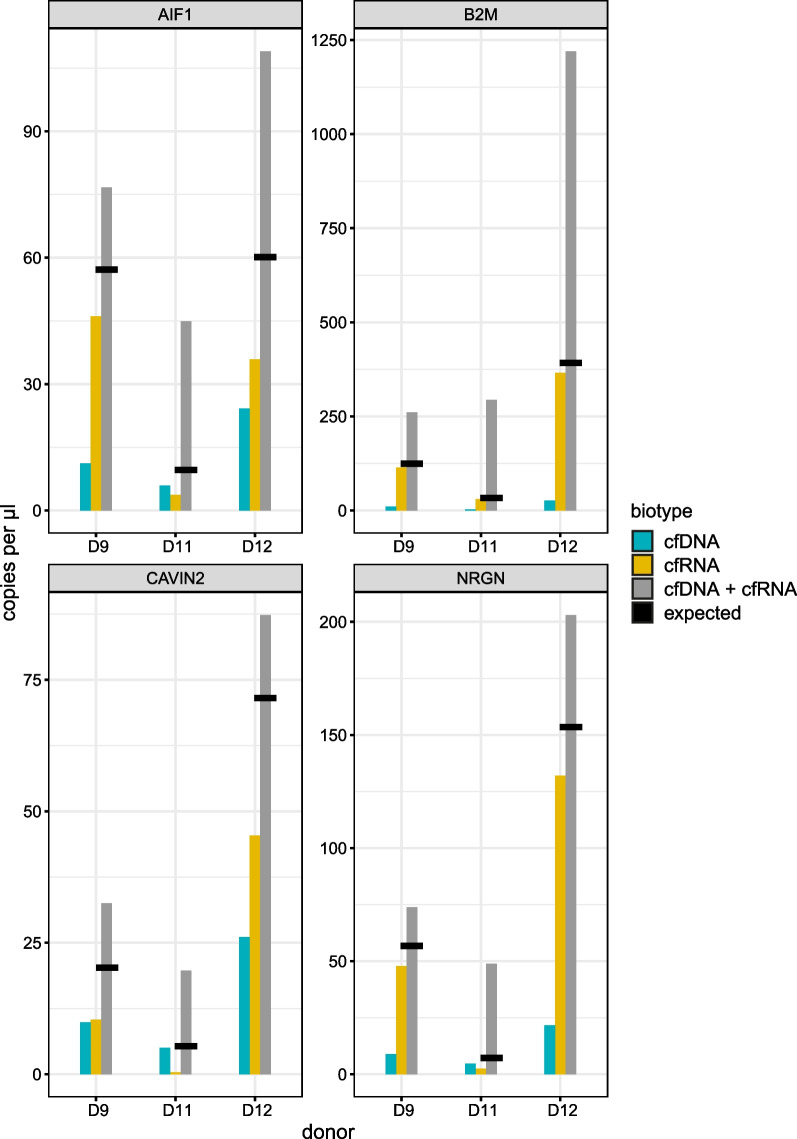
A combined quantification of nucleic acids results in higher concentrations than the quantification of cfDNA and cfRNA separately in 3 healthy donor plasma samples. Bars show concentrations for cfDNA, cfRNA and jointly measured cfDNA and cfRNA. Horizontal black lines indicate the sum of separately measured cfDNA and cfRNA (theoretical combined concentration)

Until now, we have shown that it is perfectly possible to co-purify cfDNA and cfRNA with all tested co-purification kits by quantifying the nucleic acids separately. However, jointly analyzing both cfDNA and cfRNA at the same time would improve the analytical workflow further: there is no need to split the eluate and do separate enzymatic reactions; instead, downstream analysis (e.g., dPCR quantification) can be done in one reaction, thereby saving materials, reagents and time. As a proof-of-principle, cfDNA and cfRNA was co-purified from plasma from three healthy donors (MIRA kit with 0.6 mL input volume) and quantified both separately and together. Somewhat unexpectedly, results clearly show that the jointly measured nucleic acid concentration is higher than the theoretical concentration defined as the sum of the separately measured cfDNA and cfRNA concentration (Fig. 6).

## Discussion

### Robust and reproducible workflow

We developed a robust and reproducible workflow to evaluate co-purification kit performance. First of all, DNase treatment was shown to be effective for all but one kit (MAP), despite additional purification (Vivacon columns) prior to DNase treatment to remove inhibitory reagents. Second, there is little variation between donors and collection tube types, with the exception of CAT and MAP, suggesting that co-purifications are less reproducible with these kits. Third, as expected, there is little variability between the cfDNA concentrations measured by the four assays. On the contrary, cfRNA concentrations are highly different between assays, reflecting the wide dynamic range of cfRNA abundance levels. Fourth, increasing the input volume results in a proportionally higher concentration. Fifth, quality of cfDNA was assessed to avoid measurement bias caused by cellular DNA. For 16/46 samples, cfDNA fractions could not reliably be estimated, because concentrations were below the LOD of the TapeStation or showed flat profiles. This includes all samples (*n* = 8) with 0.06 mL input volume, indicating that very little cfDNA is co-purified with such low input volume. However, concentrations could be measured with dPCR assays for both cfDNA and cfRNA, indicating the potential use of extremely low input volumes for the kits tested (MIRA, MAX), e.g., for liquid biopsy analyses in small animal studies. These results should be interpreted with caution, as these low input volumes are below the recommended input volumes by the kit’s manufacturer and cfDNA quality cannot be ascertained.

### Successful co-purification for all tested kits

By means of our optimized framework, we compared the co-purification efficiency of two manual kits (CCF, MIRA) and two semi-automated kits (MAP, CAT) with different plasma input volumes. For all samples from all kits, a concentration of cfDNA and cfRNA could be measured using the two duplex assays, indicating that all kits successfully co-purified cfDNA and cfRNA. Interestingly, in some cases, a higher eluate concentration for both cfDNA and cfRNA is obtained, by consuming less plasma (e.g., MIRA with 0.6 mL plasma input volume has at least the same eluate concentration for both cfDNA and cfRNA compared to CCF with 1 mL plasma input volume). The different co-purification kits have different eluate volumes that can be adjusted depending on the kit. This should be taken into account depending on the downstream analysis, e.g., highly concentrated eluates may be required. Of note, it is possible to further concentrate the eluate using for instance ultrafiltration columns or nucleic acid precipitation followed by resuspension in a smaller volume. For extractions with the MAPss workflow (2 mL plasma input volume), we encountered problems with the gDNA removal, as our DNase treatment was not compatible with the eluates from the MAP [15]. Extra purification of the eluates with ultrafiltration columns improved the gDNA removal efficiency, but DNA remained present in the samples. Therefore, other gDNA removal protocols should be explored in case MAPss is used for cfRNA-only applications. This example shows the importance to test DNase treatment efficiency to allow for correct quantification of cfRNA.

### Increasing detection sensitivity by co-purification

One of the clinically relevant characteristics of cfDNA and cfRNA co-purification is that it allows to increase the detection sensitivity. Detecting mutations in cancer patients’ plasma is routinely done using cfDNA, but due to low amounts in blood plasma, existing assays often face sensitivity issues. The analytical sensitivity of mutation detection can be increased by also analyzing cfRNA, at least for genes that are transcribed and for which the mRNA does not undergo non-sense mediated decay [16].

In this study, we have shown that the combined quantification of cfDNA and cfRNA results in a higher signal than their separate quantification. Remarkably, the combined quantification results in a higher concentration compared to the sum of the separate cfDNA/cfRNA concentrations, e.g., the theoretically expected concentration. There are two possible effects in the combined quantification that may in part explain the observed results: (1) no DNase treatment is done on the eluates, omitting any possible negative effects of DNase treatment on cfRNA, and (2) cDNA synthesis may improve cfDNA quantification as well. Nevertheless, these results confirm that quantifying both cfDNA and cfRNA in one single tube leads to an increased sensitivity for the assays included in this study. It is important to note that the added value of combining cfDNA and cfRNA is gene-dependent, meaning that sensitivity will only increase for genes that are sufficiently abundant in the biofluid under investigation.

## Conclusions

In this study we provide a framework for dPCR-based quantification of cfDNA and cfRNA that can be used to evaluate the performance of cfDNA/cfRNA co-purification kits. This framework was applied to two manual and two semi-automated co-purification kits. Even more, we demonstrated the added value of the combined co-purification of cfDNA and cfRNA, as combined quantification results in a higher signal compared to separate quantification. Thus, we provide strong evidence that it should be technically feasible to increase mutation detection sensitivity of specific gene targets by co-purification and quantification of cfDNA and cfRNA, which needs to be validated in future studies.

## Methods

First, we set up a framework using the dPCR technology to quantify cfDNA and cfRNA from human blood plasma in order to compare cfDNA/cfRNA co-purification kit performance. To that end, we optimized two dPCR duplex assays that are designed to quantify both cfDNA and cfRNA, by ensuring that primers and probes are located within a highly abundant exon. Next, we applied our optimized workflow to evaluate the co-purification performance of two manual and two semi-automated kits using different plasma input volumes (0.06–4 mL).

### Donor material and liquid biopsy preparation

Sample collection was approved by the ethics committee of Ghent University Hospital (registration number B670201733701) and written informed consent was obtained from the healthy donors. Venous blood was collected from healthy donors in 2 different blood collection tubes: BD Vacutainer Plastic K2EDTA tube (EDTA; Becton Dickinson and Company, 367525) and Vacuette Tube 9 mL 9NC Coagulation sodium citrate 3.2% (citrate; Greiner Bio-One, 455322). Immediately after blood draw, blood collection tubes were gently inverted five times and tubes were transported to the laboratory for immediate plasma preparation. Platelet-depleted plasma was prepared within two hours after blood draw, by means of two sequential centrifugation steps (two times 2500*g* for 15 min) on a Centrifuge 5804 (Eppendorf, 5804000013) with Rotor A-4-44 (Eppendorf, 5804709004) and appropriate adapters (Eppendorf, 5804753003). Plasma was snap frozen in liquid nitrogen and stored at − 80 °C immediately after preparation. Hemolysis was assessed by determining the absorbance at 414 nm, i.e., the levels of free heamoglobin, by spectral analysis using a NanoDrop 1000 Spectrophotometer (Thermo Fisher Scientific). Absorbances are ranging from 0.103 to 0.153.

### Cell-free nucleic acid extractions

Nucleic acids were extracted with six different commercially available (co-)purification kits by following the manufacturers’ manual: (1) miRNeasy Serum/Plasma Advanced Kit (MIRA; Qiagen, 217204), (2) QIAamp ccfDNA/RNA Kit (abbreviated to CCF; Qiagen, 55184), (3) the iCatcher Circulating cfDNA/cfRNA 4000 kit (CatchGene, AC30400) in combination with iCatcher 12 Automated Nucleic Acid Purification System (CAT; CatchGene, IC1200) (4) MagNA Pure 24 Total NA Isolation Kit with the cfNA ss 2000 and cfNA ds 2000 purification protocols (Roche, 07658036001) in combination with the MagNA Pure 24 instrument (MAP; Roche, 07290519001), (5) Maxwell ccfDNA LV Plasma Kit (Promega, AS1480) in combination with the Maxwell RSC Instrument (MAX; Promega, AS4500), and (6) miRNeasy Serum/Plasma Kit (MIR; Qiagen, 217184) (Fig. 1). The MIRA, CCF, MAP and CAT kits were included in the study to examine their capacity to co-purify cfDNA and cfRNA, while MAX and MIR kits served as a reference for cfDNA only and cfRNA only extractions, respectively, as these kits are routinely used in our department for these applications. For each kit, different plasma input volumes, ranging between 0.06 and 4 mL, from two donors (three in case of CAT) were tested with the maximum recommended elution volume (Table 1). Eluates were stored at − 80 °C until further processing.

**Table 1:** Overview of plasma input volumes and eluate volumes for each kit and protocol

Kit (purification protocol)	Plasma input volume (mL)	Eluate volume (µl)
MIRA	0.06, 0.2, 0.6	18
CCF	1, 4	18
MAX	0.06, 0.2, 1	20**
MIR	0.06, 0.2	12
MAPss (cfNA ss 2000)	2	24–32***
MAPds (cfNA ds 2000)	2	34–44***
CAT (cfDNA/cfRNA 4000)	2*, 4	17–27

*Diluted to 4 mL with PBS according to instructions of the company (CatchGene), **after vacuum centrifugation, ***after purification with Vivacon columns

Eluates of each of the four cfDNA/cfRNA co-purification kits (MIRA, CCF, MAP and CAT) were split in two equal parts, using one part for cfDNA quantification and the other part for cfRNA quantification (Fig. 1). The cfDNA part was immediately used for quantification with digital PCR without any further processing. For the cfRNA part, DNA was removed using HL-dsDNase (ArcticZymes, 70800-202) and Heat & Run 10× Reaction Buffer (ArcticZymes, 66001). Briefly, 1 µl HL-dsDNase and one tenth of the RNA input volume as reaction buffer were added, and incubated for 10 min at 37 °C, followed by 5 min inactivation at 55 °C. Subsequently, reverse transcription was performed using the iScript Advanced cDNA Synthesis Kit for RT-qPCR (Bio-Rad, 1725038) to enable digital PCR-based quantification of the cDNA (~ cfRNA). The full eluate of the MIR kit underwent DNA removal and cDNA synthesis using the same protocol, serving as a cfRNA only control. The volume of the MAX eluate was reduced to 20 µl by means of vacuum centrifugation (Eppendorf, Concentrator Plus, program V-AQ at 30 °C). As previous findings indicated incompatibilities of the MAP elution buffer for downstream DNase treatment [15], and to reduce elution volume, the MAP eluate was further purified and concentrated using Vivacon 500 2000 MWCO Hydrosart ultrafiltration columns (Sartorius, VN01H91) prior to DNA removal [15]. cDNA and cfDNA was stored at − 20 °C until further processing.

### Digital PCR assay design

For unbiased quantification of both cfDNA and cfRNA, two digital PCR duplex assays were designed each targeting a single, well covered exon of two highly abundant genes in healthy donor plasma (based on RNA sequencing data from the Extracellular RNA Quality Control study [15]): CAVIN2 (HEX)/NRGN (FAM) and AIF1 (FAM)/B2M (HEX). Designing a primer pair within a single exon allows the usage of the same assay for both cfDNA and cfRNA quantification (Table 2).

**Table 2:** Primer and probe sequences of the dPCR assays (+N is LNA nucleotide)

Target	Assay component	Sequence (5′ to 3′)	Genomic location amplicon*
NRGN	Forward primer	GTTTCTGATCTCCGTGTGT	chr11:124747135–124747204 (length: 69 bp)
	Reverse primer	CTTGGACATTCCTCTTTATTGTT	
	Probe (HEX)	TGTGACTGTGCTGGGTTGGA	
CAVIN2	Forward primer	GCACAGTTTGTTAATATTGTCTTG	chr2:191834469–191834542 (length: 73 bp)
	Reverse primer	CCTGCCTTTAGTATGAACCA	
	Probe (FAM)	ACT + CTAT + TT + GT + AA + GGTTACTT	
AIF1	Forward primer	AGCGAGAGAAAAGGAAAAGCC	chr6:31616837–31616908 (length: 71 bp)
	Reverse primer	CCTTCAAATCAGGGCAACTCA	
	Probe (FAM)	CCCCCA + GCCAAGAAAG + CTATC	
B2M	Forward primer	GTGGAGCATTCAGACTTGTCT	chr15:44715560–44715658 (length: 98 bp)
	Reverse primer	ACGGCAGGCATACTCATCTT	
	Probe (HEX)	ACA + CTGAATTCACCCCCACTGA	

*Genomic locations based on GRCh38

PCR primers were picked using the Primer3Plus tool (with default settings, except amplicon size range of 60–100 nucleotides [17]). The performance of the primers was thoroughly evaluated in silico. To determine the primer specificity, BiSearch e-PCR (with default settings, except for mismatch string: 1233333333333333 [18]) and the UCSC tool [19] were used. Subsequently, the OligoEvaluator tool [20] was used to check for secondary structure formation and GC content. Lastly, the SBT tool [21] was used to predict the melting temperature (Tm). The hydrolysis probe sequence was picked manually in between forward and reverse primer, aiming for a Tm of at least 3 °C higher than the primers’ Tm. Primers and probes were ordered with Integrated DNA Technologies (IDT, Leuven, Belgium). Probes were ordered as double-quenched hydrolysis probes with optional LNA nucleotides to enhance the Tm and purified by HPLC. Primers were purified by standard desalting. All oligonucleotides were resuspended in TE buffer (10 mM Tris–HCl (pH 8.0), 0.1 mM EDTA) to 100 µM (primers) and 10 µM (probes) and stored at − 20 °C. The primer efficiency and specificity was also validated on a dilution series of Quantitative PCR Human Reference Total RNA (Agilent technologies, 750500), reverse transcribed to cDNA.

### Quantification of cfDNA and cfRNA using dPCR

Digital PCR was performed using the QX100 Droplet Digital PCR system (Bio-Rad, California, USA), according to the manufacturer’s protocol with minor modifications (250 nM primer and 100 nM probe reaction mix concentration). Each 20 µl dPCR reaction contains 10 µl 2 × ddPCR Supermix for Probes (Bio-Rad, 1863010), 2 µl of primer and probe mix (with a total of 4 primers and 2 probes per duplex assay) and 2—8 µl template. After pipetting 20 µl sample mix and 70 µl droplet generation oil (Bio-Rad, 1863005) in the cartridge (Bio-Rad, 1864008), droplets were generated by means of the Bio-Rad QX100 Droplet Generator. Droplets were then transferred from the cartridge to a 96-well plate (Bio-Rad, 12001925) and a thermocycling program was performed on a C1000 Touch Thermal cycler (Bio-Rad): 95 °C for 10 min, followed by 40 cycles of 15 s on 95 °C and 1 min at 56.9 °C (optimized annealing temperature for both duplexes by means of a gradient dPCR). Finally, reactions were heated to 98 °C for 10 min and then cooled down to 12 °C before transferring the plate to the QX100 Droplet Reader (Bio-Rad). Each plate also included a positive control (PC) and negative no template control (NTC). QuantaSoft Analysis Pro Software Version 1.3.2.0 was used to calculate the number of copies per µl in the dPCR reaction by manual thresholding, followed by copies per µl eluate concentration determination (Additional file 6: Fig. S3) to enable comparison among kits.

### Combined quantification of cfDNA and cfRNA

To determine the added value of the combined analysis of cfDNA and cfRNA, separate quantification of cfDNA and cfRNA was compared to combined quantification of both. As this is a proof-of-principle experiment, only one kit (MIRA0.06) was used to co-purify nucleic acids from plasma of three healthy donors (platelet-free plasma). Eluates were split into three equal parts to quantify cfDNA, cfRNA and cfDNA/cfRNA using the two dPCR duplex assays (Additional file 7: Fig. S4). For cfDNA/cfRNA combined quantification, only cDNA synthesis was performed on the eluate, as such containing both cfDNA and cDNA, while for cfRNA only quantification, a DNase treatment was performed prior to cDNA synthesis. For cfDNA quantification, the eluate remained untouched.

## Supplementary Information

**Additional file 1: Figure S1**. Efficiency of DNase treatment after cfDNA/cfRNA (co-)purification. Efficiency was assessed with the NRGN assay only. Error bars indicate standard error. Quantifications for CAT2, MAPss2 and MAPds2 are based on one replicate.

**Additional file 2: Table S1**. Additional kit characteristics and remarks

**Additional file 3: Table S2**. Comparison of expected and measured cfDNA or cfRNA concentration ratio, based on plasma input volume, within a given nucleic acid extraction kit.

**Additional file 4: Table S3**. Concentration (geometric mean of four assays) and yield (concentration multiplied by eluate volume) in the eluates from the kits with the different tested input volumes. Relative concentrations and yield were determined by rescaling to the highest value.

**Additional file 5: Figure S2**. Overview of TapeStation results. Results from TapeStation of all cfDNA samples purified with one of the six (co-)purification kits. There are 13/46 samples that have concentrations below LOD (20 pg/µl), and 2/46 samples without any peaks (flat profiles). For 1/46 samples there was not enough material left for TapeStation.

**Additional file 6: Figure S3**. Detailed description of laboratory workflow. Workflow used to determine co-purification performance of each kit and subsequent calculations to determine the concentrations in each eluate: (A) MIRA and CCF, (B) MIR, (C) CAT, (D) MAX, and (E) MAP.

**Additional file 7: Figure S4**. Experimental overview of a combined cfDNA/cfRNA quantification and a parallel quantification of cfDNA and cfRNA.

## Data Availability

The raw data are available on reasonable request from the corresponding author.

## Abbreviations

CAT: iCatcher Circulating cfDNA/cfRNA 4000 kit
CCF: QIAamp ccfDNA/RNA Kit
cfDNA: Cell-free DNA
cfRNA: Cell-free RNA
dPCR: Digital PCR
EVs: Extracellular vesicles
HMW: High molecular weight
MAPss: MagNA Pure 24 Total NA Isolation Kit with the cfNA ss 2000
MAPds: MagNA Pure 24 Total NA Isolation Kit with the cfNA ds 2000
MAX: Maxwell ccfDNA LV Plasma Kit
MIR: miRNeasy Serum/Plasma Kit
MIRA: miRNeasy Serum/Plasma Advanced kit
