# Supplementary figures and images for: Digital PCR-based evaluation of nucleic acid extraction kit performance for the co-purification of cell-free DNA and RNA
Source: Hum Genomics. 2022 Dec 31;16:73. doi: 10.1186/s40246-022-00446-4 (PMC9805675; doi:10.1186/s40246-022-00446-4)

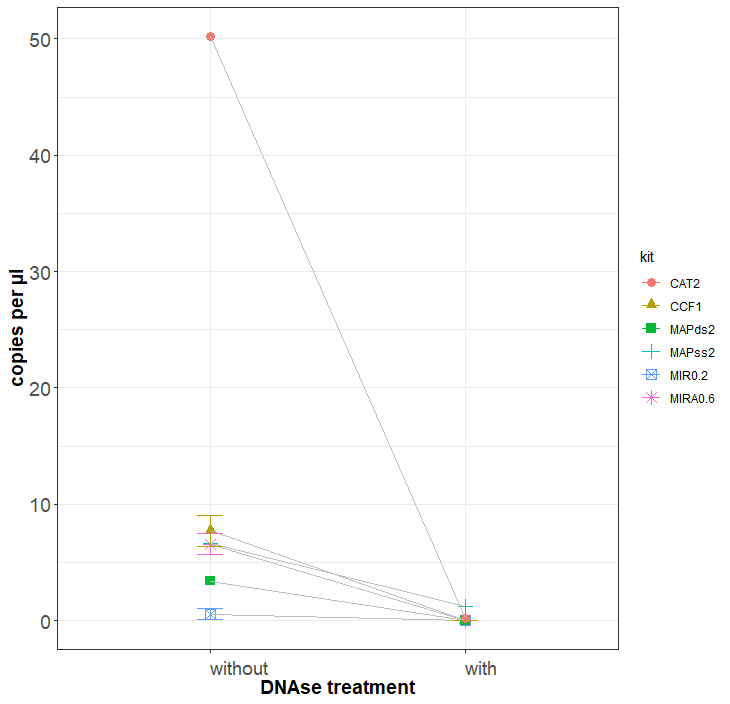

Supplement: Supplementary file 1 — Additional file 1: Figure S1. Efficiency of DNase treatment after cfDNA/cfRNA (co-)purification. Efficiency was assessed with the NRGN assay only. Error bars indicate standard error. Quantifications for CAT2, MAPss2 and MAPds2 are based on one replicate. [file 40246_2022_446_MOESM1_ESM.png]

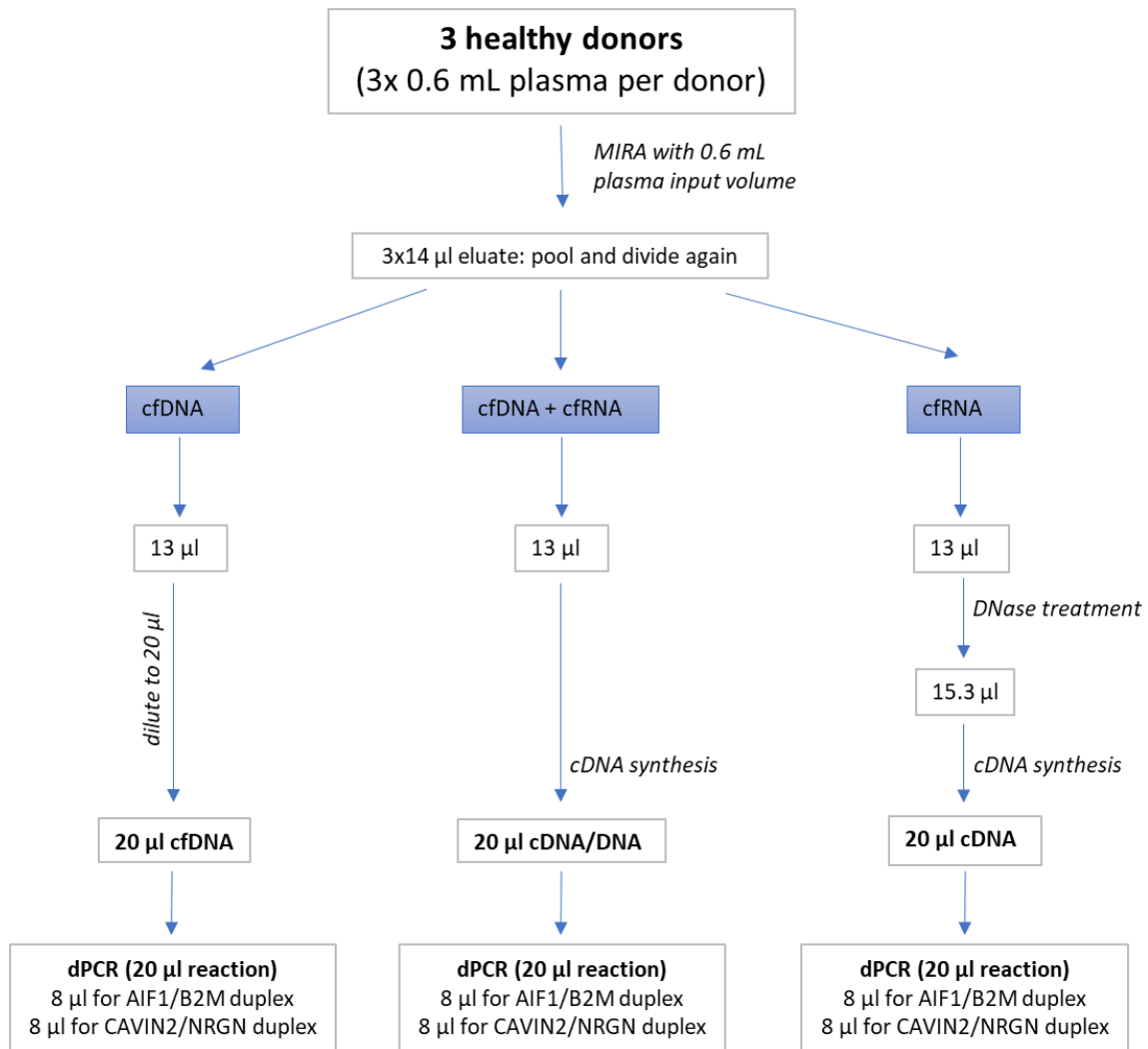

Supplement: Supplementary file 7 — Additional file 7: Figure S4. Experimental overview of a combined cfDNA/cfRNA quantification and a parallel quantification of cfDNA and cfRNA. [file 40246_2022_446_MOESM7_ESM.pdf]
