# Supplementary material for: Digital PCR-based evaluation of nucleic acid extraction kit performance for the co-purification of cell-free DNA and RNA
Source: Hum Genomics. 2022 Dec 31;16:73. doi: 10.1186/s40246-022-00446-4 (PMC9805675; doi:10.1186/s40246-022-00446-4)
Supplement: Supplementary file 5 — Additional file 5: Figure S2. Overview of TapeStation results. Results from TapeStation of all cfDNA samples purified with one of the six (co-)purification kits. There are 13/46 samples that have concentrations below LOD (20 pg/µl), and 2/46 samples without any peaks (flat profiles). For 1/46 samples there was not enough material left for TapeStation. [file 40246_2022_446_MOESM5_ESM.pdf]

D1_EDTA_CCF_1

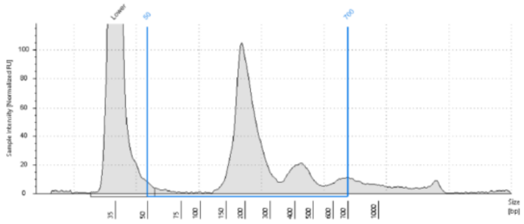

Region Table

From [bp]	To [bp]	Average Size [bp]	Conc. [pg/µl]	Region Molarity [pmol/l]	%of Total	Region Comment	Color
50	700	271	147	1090	85.67	%cfDNA	

D1_EDTA_CCF_4

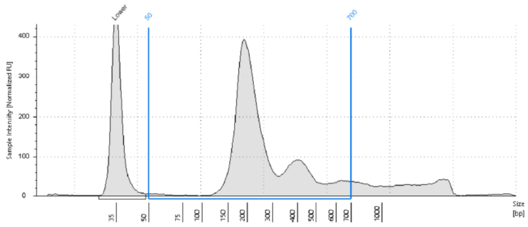

Region Table

From [bp]	To [bp]	Average Size [bp]	Conc. [pg/µl]	Region Molarity [pmol/l]	%of Total	Region Comment	Color
50	700	275	689	4650	82.62	%cfDNA	

D1_EDTA_MIRA_0.06 (<LOD)

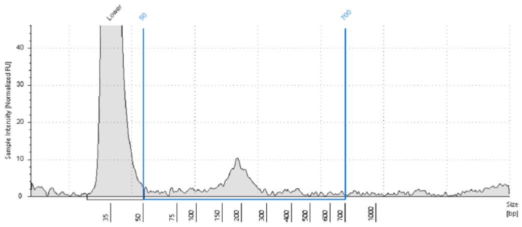

Region Table

From [bp]	To [bp]	Average Size [bp]	Conc. [pg/µl]	Region Molarity [pmol/l]	%of Total	Region Comment	Color
50	700	213	16.4	211	74.00	%cfDNA	

D1_EDTA_MIRA_0.2

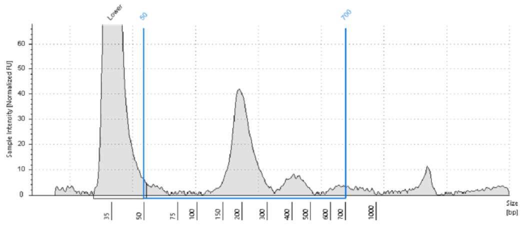

Region Table

From [bp]	To [bp]	Average Size [bp]	Conc. [pg/µl]	Region Molarity [pmol/l]	%of Total	Region Comment	Color
50	700	245	55.1	485	81.13	%cfDNA	

D1_EDTA_MIRA_0.6

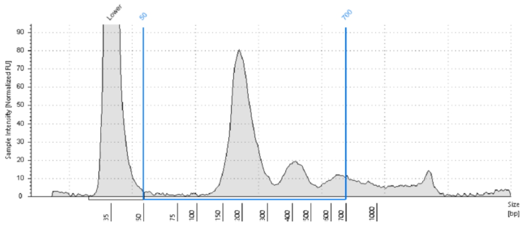

Region Table

From [bp]	To [bp]	Average Size [bp]	Conc. [pg/µl]	Region Molarity [pmol/l]	%of Total	Region Comment	Color
50	700	278	137	971	80.28	%cfDNA	

D1_CITR_CCF_1

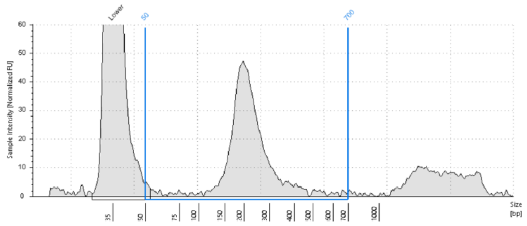

Region Table

From [bp]	To [bp]	Average Size [bp]	Conc. [pg/µl]	Region Molarity [pmol/l]	%of Total	Region Comment	Color
50	700	231	72.3	601	70.30	%cfDNA	

D1_CITR_CCF_4

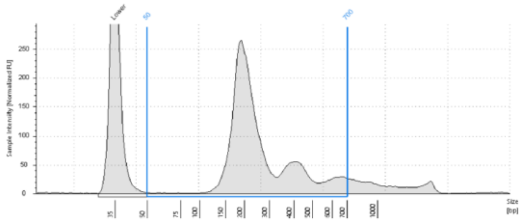

Region Table

From [bp]	To [bp]	Average Size [bp]	Conc. [pg/µl]	Region Molarity [pmol/l]	%of Total	Region Comment	Color
50	700	273	456	3070	86.77	%cfDNA	

D1_CITR_MIRA_0.06 (<LOD)

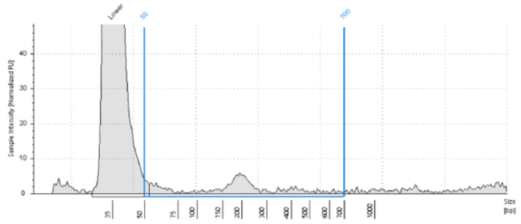

Region Table

From [bp]	To [bp]	Average Size [bp]	Conc. [pg/µl]	Region Molarity [pmol/l]	%of Total	Region Comment	Color
50	700	227	7.80	150	62.04	%cfDNA	

D1_CITR_MIRA_0.2

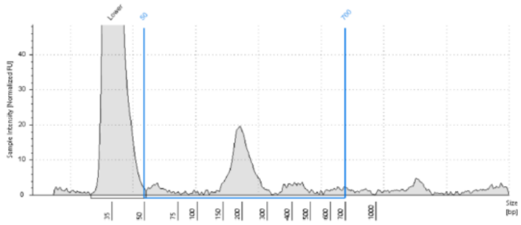

Region Table

From [bp]	To [bp]	Average Size [bp]	Conc. [pg/µl]	Region Molarity [pmol/l]	%of Total	Region Comment	Color
50	700	241	27.0	262	75.63	%cfDNA	

D1_CITR_MIRA_0.6

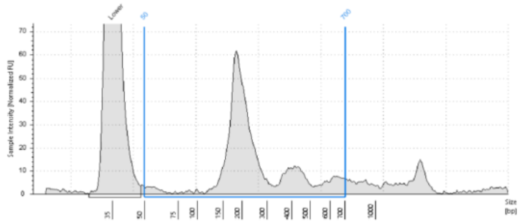

Region Table

From [bp]	To [bp]	Average Size [bp]	Conc. [pg/µl]	Region Molarity [pmol/l]	%of Total	Region Comment	Color
50	700	266	93.0	717	77.85	%cfDNA	

D2_EDTA_CCF_1

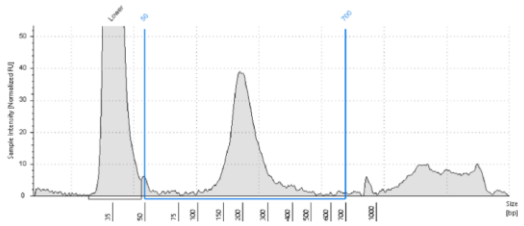

Region Table

From [bp]	To [bp]	Average Size [bp]	Conc. [pg/µl]	Region Molarity [pmol/l]	% of Total	Region Comment	Color
50	700	221	59.0	510	63.74	%cfDNA	

D2_EDTA_CCF_4

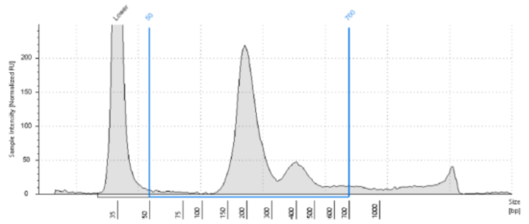

Region Table

From [bp]	To [bp]	Average Size [bp]	Conc. [pg/µl]	Region Molarity [pmol/l]	% of Total	Region Comment	Color
50	700	260	323	2320	84.36	%cfDNA	

D2_EDTA_MIRA_0.06 (<LOD)

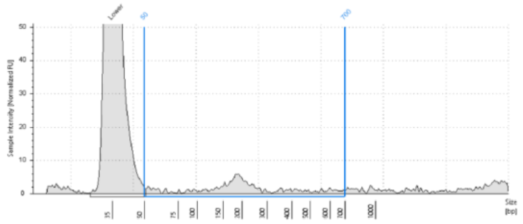

Region Table

From [bp]	To [bp]	Average Size [bp]	Conc. [pg/µl]	Region Molarity [pmol/l]	% of Total	Region Comment	Color
50	700	242	10.8	141	55.79	%cfDNA	

D2_EDTA_MIRA_0.2 (<LOD)

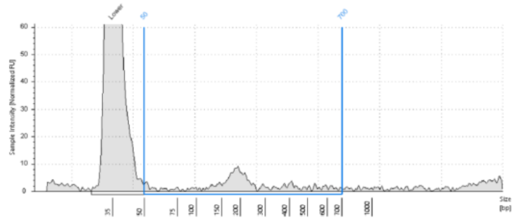

Region Table

From [bp]	To [bp]	Average Size [bp]	Conc. [pg/µl]	Region Molarity [pmol/l]	% of Total	Region Comment	Color
50	700	253	15.7	181	62.57	%cfDNA	

D2_EDTA_MIRA_0.6

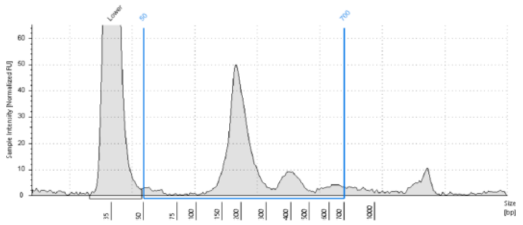

Region Table

From [bp]	To [bp]	Average Size [bp]	Conc. [pg/µl]	Region Molarity [pmol/l]	% of Total	Region Comment	Color
50	700	250	67.4	555	84.25	%cfDNA	

D2_CITR_CCF_1

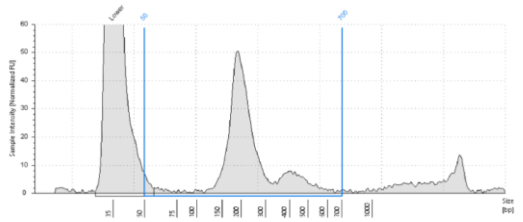

Region Table

From [bp]	To [bp]	Average Size [bp]	Conc. [pg/µl]	Region Molarity [pmol/l]	% of Total	Region Comment	Color
50	700	243	68.3	576	78.86	%cfDNA	

D2_CITR_CCF_4

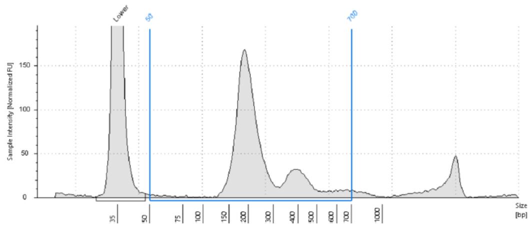

Region Table

From [bp]	To [bp]	Average Size [bp]	Conc. [pg/µl]	Region Molarity [pmol/l]	% of Total	Region Comment	Color
50	700	252	254	1850	83.49	%cfDNA	

D2_CITR_MIRA_0.06 (<LOD)

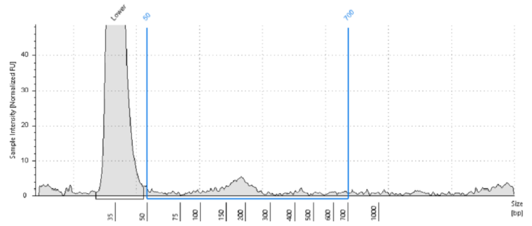

Region Table

From [bp]	To [bp]	Average Size [bp]	Conc. [pg/µl]	Region Molarity [pmol/l]	% of Total	Region Comment	Color
50	700	231	11.4	148	60.08	%cfDNA	

D2_CITR_MIRA_0.2

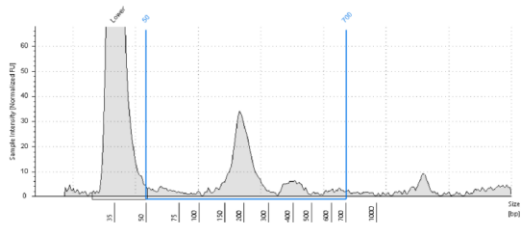

Region Table

From [bp]	To [bp]	Average Size [bp]	Conc. [pg/µl]	Region Molarity [pmol/l]	% of Total	Region Comment	Color
50	700	234	41.8	428	79.41	%cfDNA	

D2_CITR_MIRA_0.6

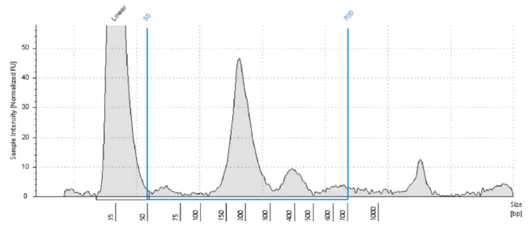

Region Table

From [bp]	To [bp]	Average Size [bp]	Conc. [pg/µl]	Region Molarity [pmol/l]	% of Total	Region Comment	Color
50	700	243	65.3	563	80.19	%cfDNA	

D1_EDTA_MAX_0.06 (<LOD)

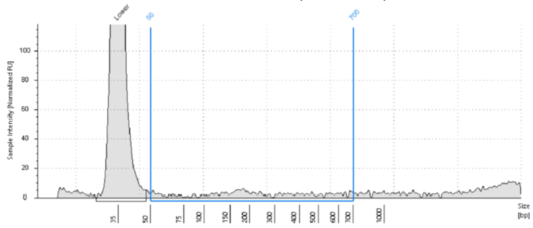

Region Table

From [bp]	To [bp]	Average Size [bp]	Conc. [pg/µl]	Region Molarity [pmol/l]	%of Total	Region Comment	Color
50	700	280	15.0	212	34.80	%cfDNA	

D1_EDTA_MAX_0.2

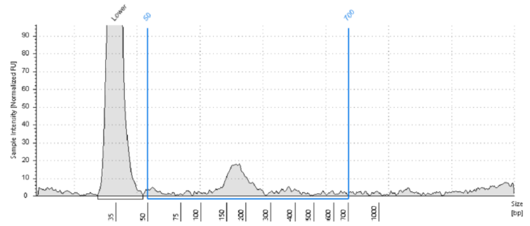

Region Table

From [bp]	To [bp]	Average Size [bp]	Conc. [pg/µl]	Region Molarity [pmol/l]	%of Total	Region Comment	Color
50	700	221	29.7	374	65.29	%cfDNA	

D1_EDTA_MAX_1

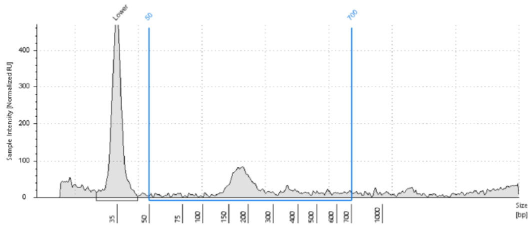

Region Table

From [bp]	To [bp]	Average Size [bp]	Conc. [pg/µl]	Region Molarity [pmol/l]	%of Total	Region Comment	Color
50	700	261	138	1510	64.82	%cfDNA	

D1_CITR_MAX_0.06 (<LOD)

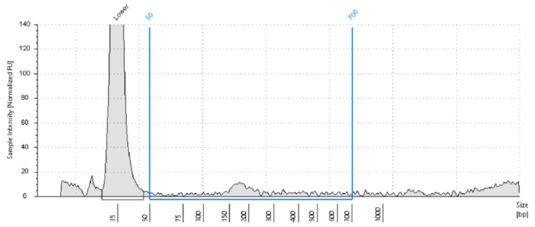

Region Table

From [bp]	To [bp]	Average Size [bp]	Conc. [pg/µl]	Region Molarity [pmol/l]	%of Total	Region Comment	Color
50	700	261	18.8	278	42.69	%cfDNA	

D1_CITR_MAX_0.2

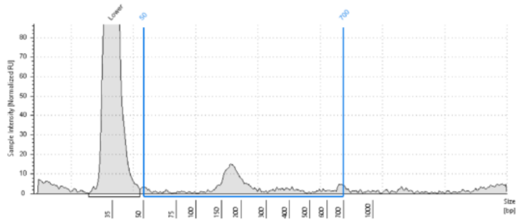

Region Table

From [bp]	To [bp]	Average Size [bp]	Conc. [pg/µl]	Region Molarity [pmol/l]	%of Total	Region Comment	Color
50	700	255	22.5	236	69.20	%cfDNA	

D1_CITR_MAX_1

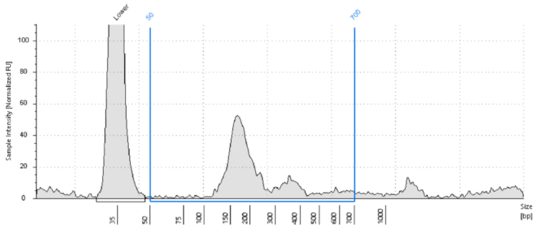

Region Table

From [bp]	To [bp]	Average Size [bp]	Conc. [pg/µl]	Region Molarity [pmol/l]	%of Total	Region Comment	Color
50	700	241	88.1	761	76.99	%cfDNA	

D2_EDTA_MAX_0.06 (flat profile)

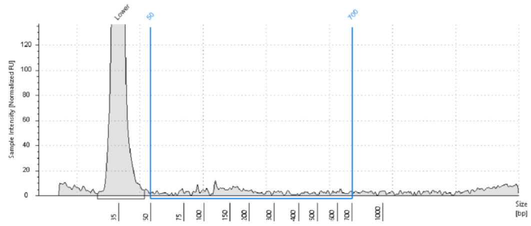

Region Table

From [bp]	To [bp]	Average Size [bp]	Conc. [pg/µl]	Region Molarity [pmol/l]	%of Total	Region Comment	Color
50	700	242	21.3	307	47.51	%cfDNA	

D2_EDTA_MAX_0.2 (flat profile)

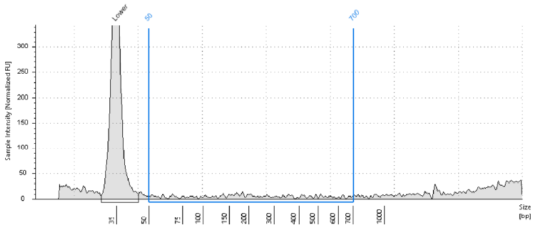

Region Table

From [bp]	To [bp]	Average Size [bp]	Conc. [pg/µl]	Region Molarity [pmol/l]	%of Total	Region Comment	Color
50	700	260	28.0	530	21.42	%cfDNA	

D2_EDTA_MAX_1

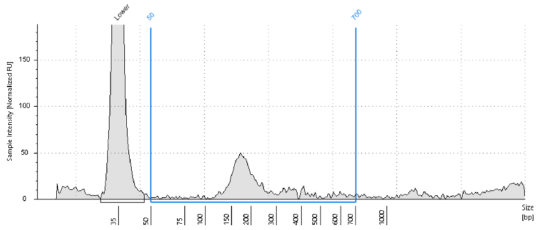

Region Table

From [bp]	To [bp]	Average Size [bp]	Conc. [pg/µl]	Region Molarity [pmol/l]	%of Total	Region Comment	Color
50	700	243	73.1	723	66.62	%cfDNA	

D2_CITR_MAX_0.06 (<LOD)

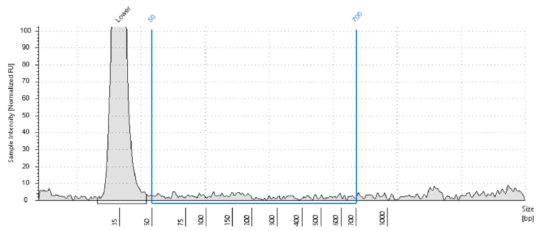

Region Table

From [bp]	To [bp]	Average Size [bp]	Conc. [pg/µl]	Region Molarity [pmol/l]	%of Total	Region Comment	Color
50	700	239	11.9	272	37.58	%cfDNA	

D2_CITR_MAX_0.2 (<LOD)

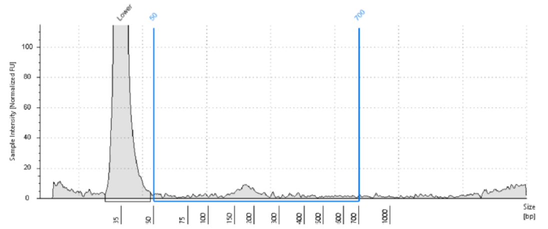

Region Table

From [bp]	To [bp]	Average Size [bp]	Conc. [pg/µl]	Region Molarity [pmol/l]	% of Total	Region Comment	Color
50	700	231	4.71	235	37.78	%cfDNA	

D2_CITR_MAX_1

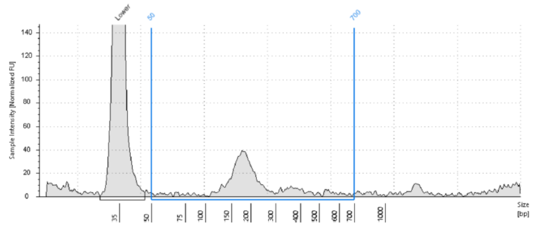

Region Table

From [bp]	To [bp]	Average Size [bp]	Conc. [pg/µl]	Region Molarity [pmol/l]	% of Total	Region Comment	Color
50	700	232	57.9	662	73.79	%cfDNA	

D1_CITR_MAPss_2 (<LOD)

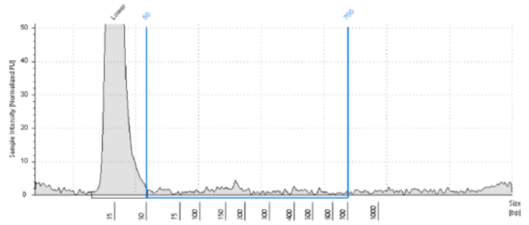

Region Table

From [bp]	To [bp]	Average Size [bp]	Conc. [pg/µl]	Region Molarity [pmol/l]	% of Total	Region Comment	Color
50	700	234	4.00	124	44.60	%cfDNA	

D2_CITR_MAPss_2 (<LOD)

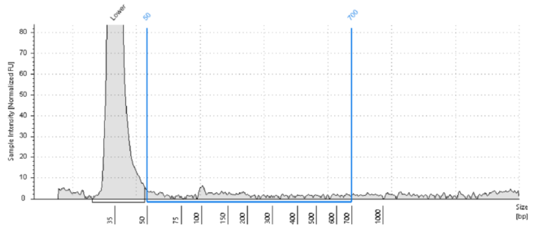

Region Table

From [bp]	To [bp]	Average Size [bp]	Conc. [pg/µl]	Region Molarity [pmol/l]	% of Total	Region Comment	Color
50	700	233	3.46	186	48.45	%cfDNA	

D1_EDTA_MAPss_2 (<LOD)

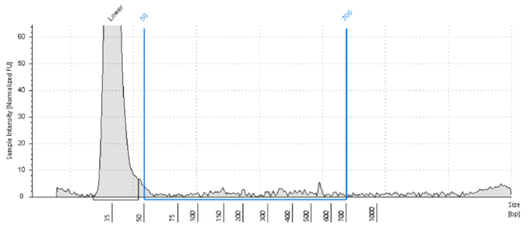

Region Table

From [bp]	To [bp]	Average Size [bp]	Conc. [pg/µl]	Region Molarity [pmol/l]	% of Total	Region Comment	Color
50	700	290	5.35	116	43.69	%cfDNA	

D2_EDTA_MAPss_2 (<LOD)

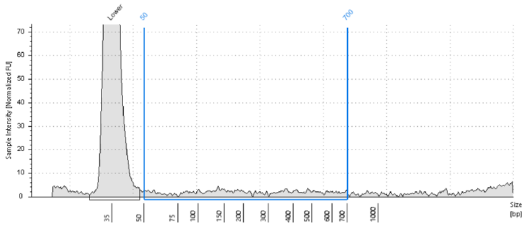

Region Table

From [bp]	To [bp]	Average Size [bp]	Conc. [pg/µl]	Region Molarity [pmol/l]	% of Total	Region Comment	Color
50	700	275	8.80	192	51.36	%cfDNA	

D1_CITR_MAPds_2

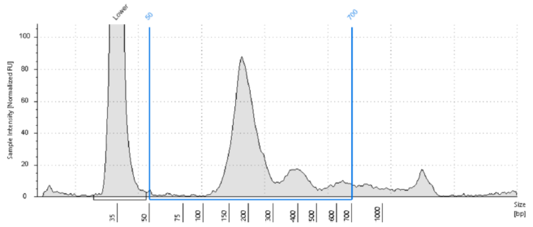

Region Table

From [bp]	To [bp]	Average Size [bp]	Conc. [pg/µl]	Region Molarity [pmol/l]	% of Total	Region Comment	Color
50	700	258	150	1170	85.51	%cfDNA	

D2_CITR_MAPds_2

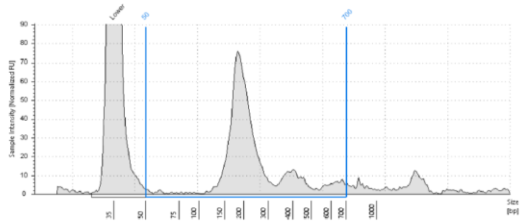

Region Table

From [bp]	To [bp]	Average Size [bp]	Conc. [pg/µl]	Region Molarity [pmol/l]	% of Total	Region Comment	Color
50	700	253	109	865	86.37	%cfDNA	

D1_EDTA_MAPds_2

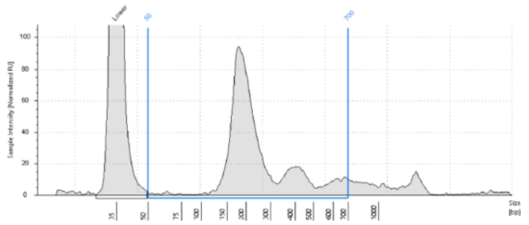

Region Table

From [bp]	To [bp]	Average Size [bp]	Conc. [pg/µl]	Region Molarity [pmol/l]	% of Total	Region Comment	Color
50	700	263	155	1160	85.61	%cfDNA	

D2_EDTA_MAPds_2

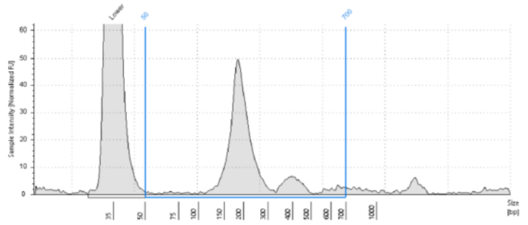

Region Table

From [bp]	To [bp]	Average Size [bp]	Conc. [pg/µl]	Region Molarity [pmol/l]	% of Total	Region Comment	Color
50	700	235	58.4	491	89.46	%cfDNA	

D1(PLA016740)_EDTA_CAT_4

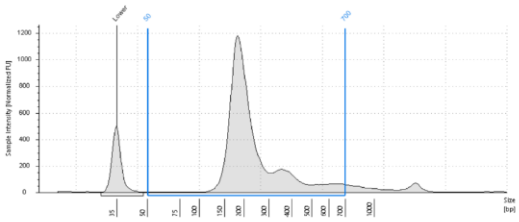

Region Table

From [bp]	To [bp]	Average Size [bp]	Conc. [pg/µl]	Region Molarity [pmol/l]	% of Total	Region Comment	Color
50	700	247	1790	13000	92.46	%cfDNA	

D2(PLA016741)_EDTA_CAT_4

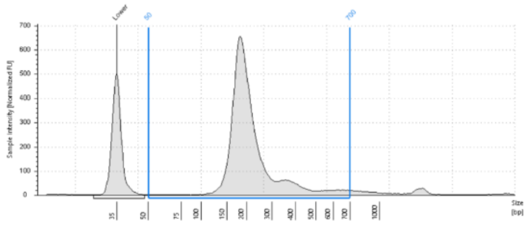

Region Table

From [bp]	To [bp]	Average Size [bp]	Conc. [pg/µl]	Region Molarity [pmol/l]	% of Total	Region Comment	Color
50	700	229	891	6720	94.32	%cfDNA	

D3(PLA016768)_EDTA_CAT_4

Not enough material left for TapeStation

D1(PLA016740)_EDTA_CAT_2

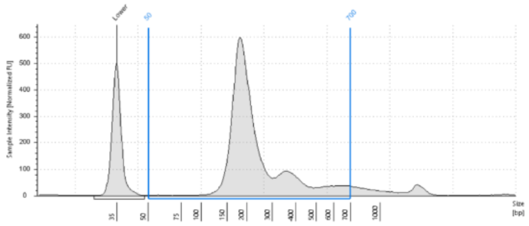

Region Table

From [bp]	To [bp]	Average Size [bp]	Conc. [pg/µl]	Region Molarity [pmol/l]	% of Total	Region Comment	Color
50	700	250	940	6770	90.65	%cfDNA	

D2(PLA016741)_EDTA_CAT_2

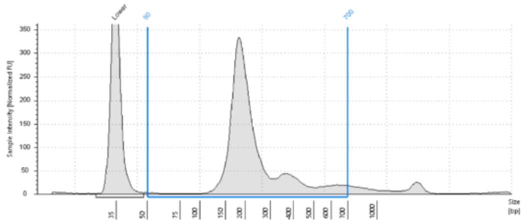

Region Table

From [bp]	To [bp]	Average Size [bp]	Conc. [pg/µl]	Region Molarity [pmol/l]	% of Total	Region Comment	Color
50	700	245	489	3580	90.71	%cfDNA	

D3(PLA016768)_EDTA_CAT_2

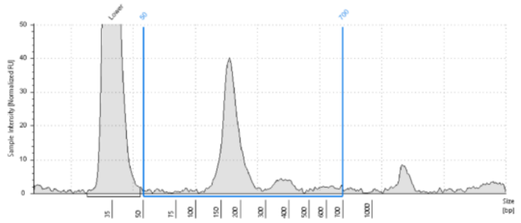

Region Table

From [bp]	To [bp]	Average Size [bp]	Conc. [pg/µl]	Region Molarity [pmol/l]	% of Total	Region Comment	Color
50	700	220	48.9	433	77.96	%cfDNA	
