# Supplementary material for: Digital PCR-based evaluation of nucleic acid extraction kit performance for the co-purification of cell-free DNA and RNA
Source: Hum Genomics. 2022 Dec 31;16:73. doi: 10.1186/s40246-022-00446-4 (PMC9805675; doi:10.1186/s40246-022-00446-4)
Supplement: Supplementary file 6 — Additional file 6: Figure S3. Detailed description of laboratory workflow. Workflow used to determine co-purification performance of each kit and subsequent calculations to determine the concentrations in each eluate: (A) MIRA and CCF, (B) MIR, (C) CAT, (D) MAX, and (E) MAP. [file 40246_2022_446_MOESM6_ESM.pdf]

A

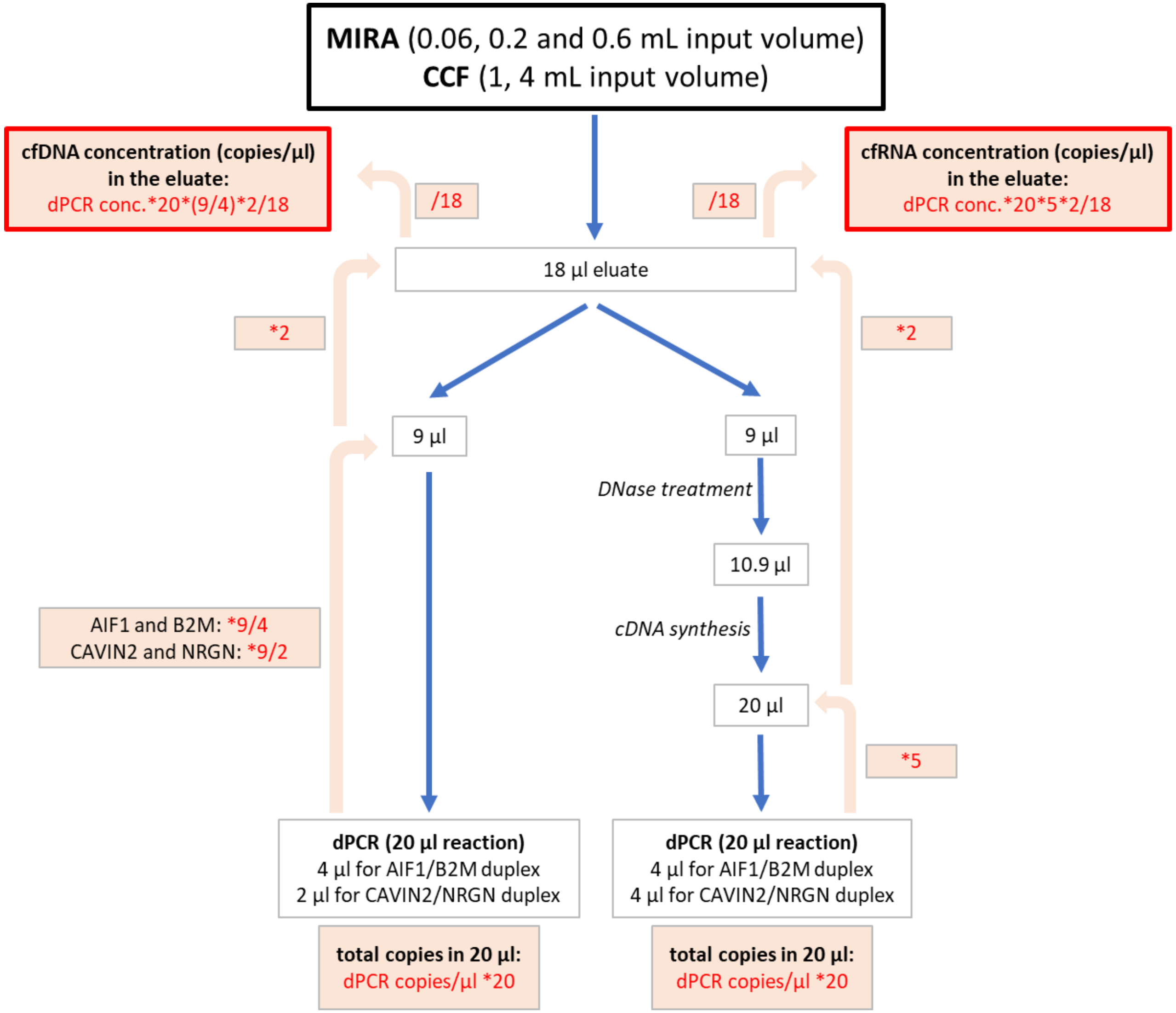

MIRA (0.06, 0.2 and 0.6 mL input volume)
CCF (1, 4 mL input volume)
cfDNA concentration (copies/µl) in the eluate:
dPCR conc.*20*(9/4)*2/18
cfRNA concentration (copies/µl) in the eluate:
dPCR conc.*20*5*2/18
/18
/18
18 µl eluate
*2
*2
9 µl
9 µl
DNase treatment
10.9 µl
cDNA synthesis
20 µl
*5
AIF1 and B2M: *9/4
CAVIN2 and NRGN: *9/2
dPCR (20 µl reaction)
4 µl for AIF1/B2M duplex
2 µl for CAVIN2/NRGN duplex
dPCR (20 µl reaction)
4 µl for AIF1/B2M duplex
4 µl for CAVIN2/NRGN duplex
total copies in 20 µl:
dPCR copies/µl *20
total copies in 20 µl:
dPCR copies/µl *20

B

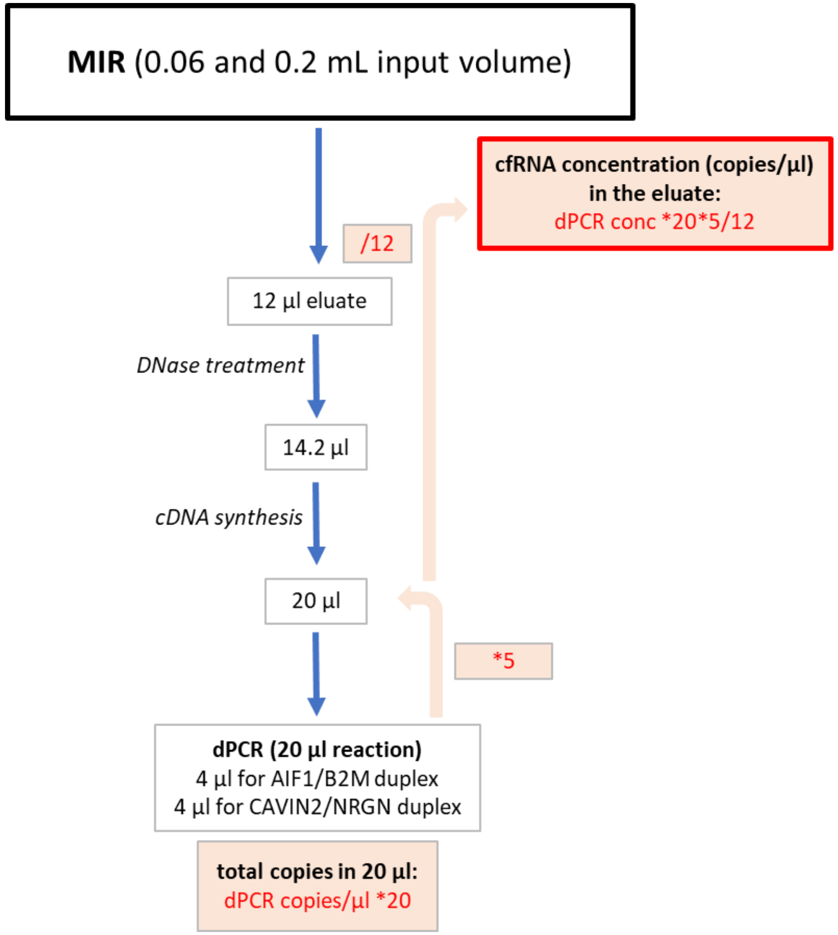
MIR (0.06 and 0.2 mL input volume)
/12
12 µl eluate
DNase treatment
14.2 µl
cDNA synthesis
20 µl
*5
dPCR (20 µl reaction)
4 µl for AIF1/B2M duplex
4 µl for CAVIN2/NRGN duplex
total copies in 20 µl:
dPCR copies/µl *20
cfRNA concentration (copies/µl) in the eluate:
dPCR conc *20*5/12

C

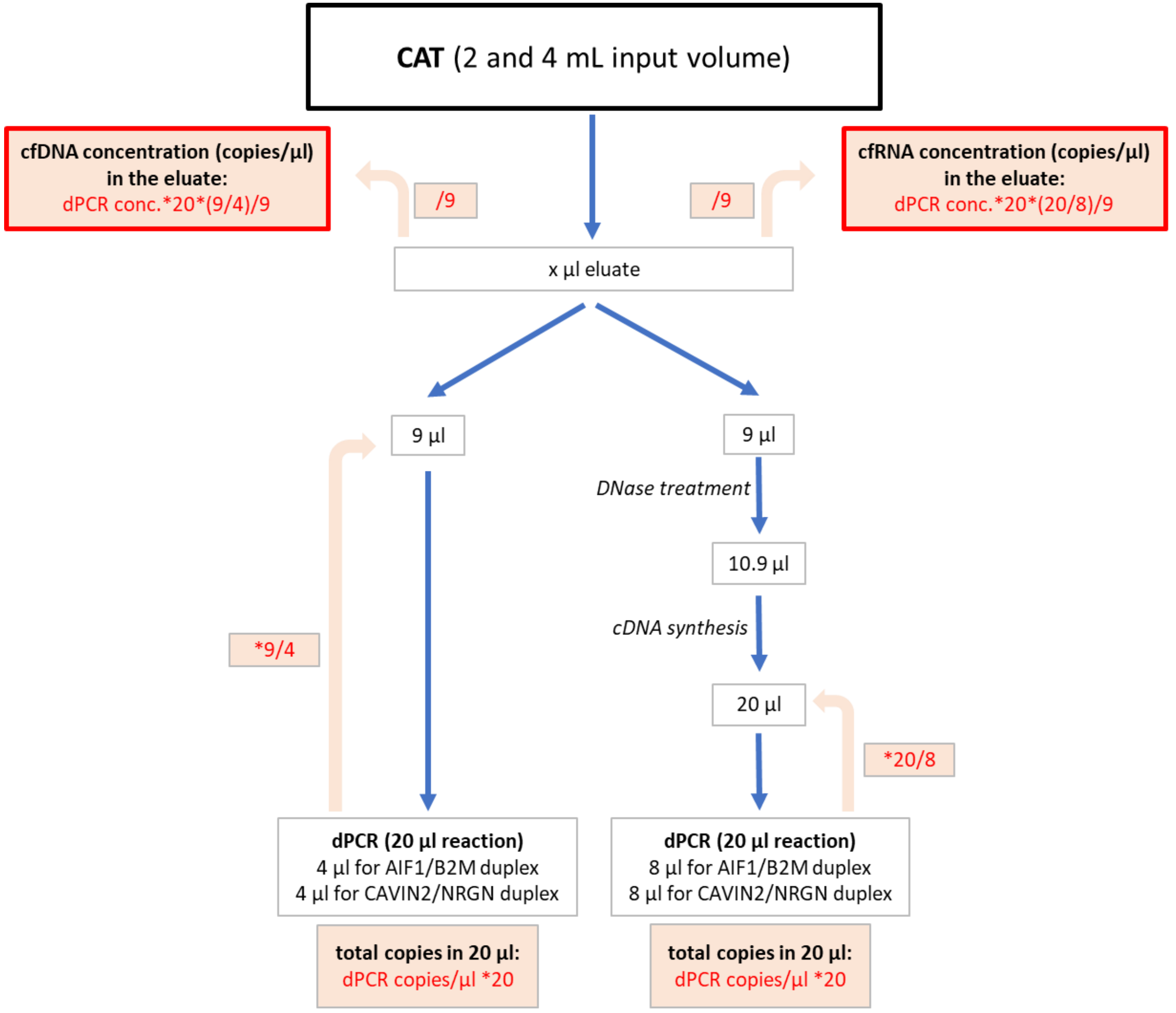

CAT2	
**sample**	**x**
D1 (EDTA)	19 µl
D2 (EDTA)	19 µl
D3 (EDTA)	27 µl

CAT4	
**sample**	**x**
D1 (EDTA)	21 µl
D2 (EDTA)	23 µl
D3 (EDTA)	18 µl

D

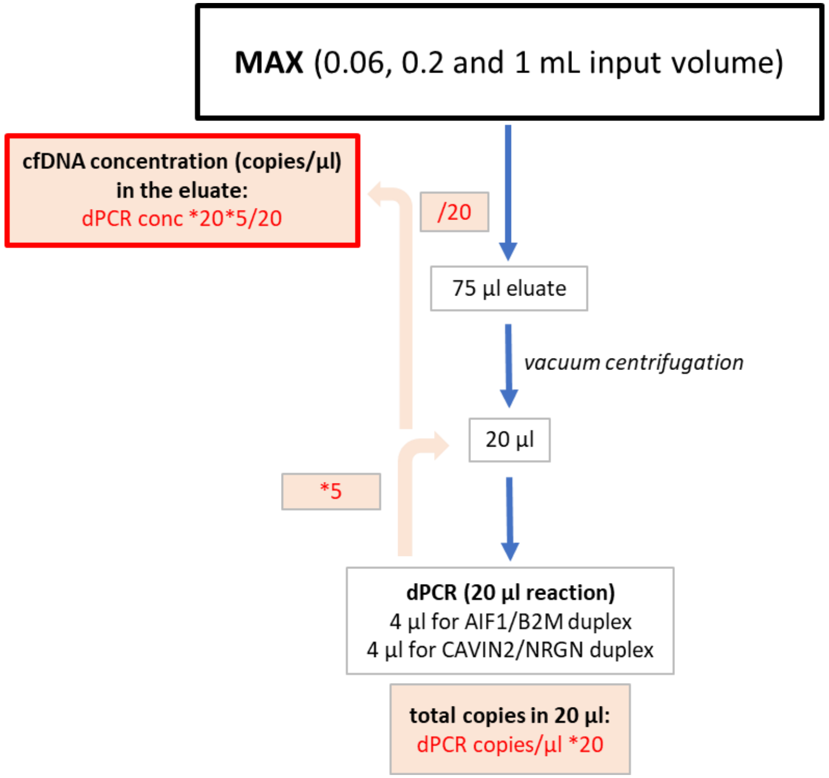

MAX (0.06, 0.2 and 1 mL input volume)
cfDNA concentration (copies/µl)
in the eluate:
dPCR conc *20*5/20
/20
75 µl eluate
vacuum centrifugation
20 µl
*5
dPCR (20 µl reaction)
4 µl for AIF1/B2M duplex
4 µl for CAVIN2/NRGN duplex
total copies in 20 µl:
dPCR copies/µl *20

E

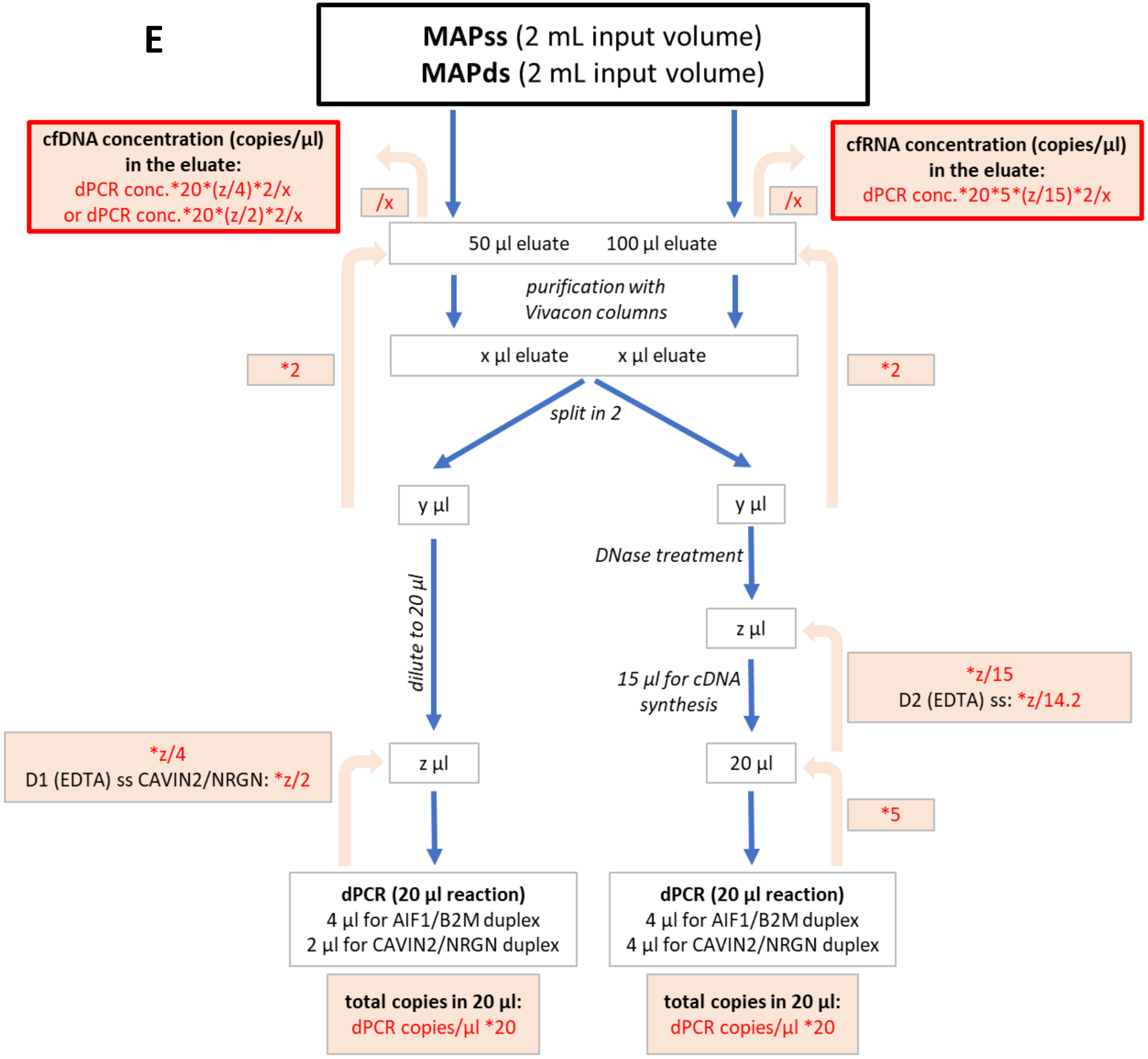

MAPss		MAPds	
**sample**	**x**	**sample**	**x**
D1 (citrate)	32 µl	D1 (citrate)	34 µl
D2 (citrate)	32 µl	D2 (citrate)	36 µl
D1 (EDTA)	30 µl	D1 (EDTA)	42 µl
D2 (EDTA)	24 µl	D2 (EDTA)	44 µl

cfDNA		cfRNA	
**sample**	**y**	**sample**	**y**
D1 (citrate) ss	16 µl	D1 (citrate) ss	16 µl
D2 (citrate) ss	16 µl	D2 (citrate) ss	16 µl
D1 (EDTA) ss	15 µl	D1 (EDTA) ss	15 µl
D2 (EDTA) ss	12 µl	D2 (EDTA) ss	12 µl
D1 (citrate) ds	17 µl	D1 (citrate) ds	17 µl
D2 (citrate) ds	18 µl	D2 (citrate) ds	18 µl
D1 (EDTA) ds	21 µl	D1 (EDTA) ds	21 µl
D2 (EDTA) ds	22 µl	D2 (EDTA) ds	22 µl

cfDNA		cfRNA	
**sample**	**z**	**sample**	**z**
D1 (citrate) ss	20 µl	D1 (citrate) ss	18.6 µl
D2 (citrate) ss	20 µl	D2 (citrate) ss	18.6 µl
D1 (EDTA) ss	20 µl	D1 (EDTA) ss	17.5 µl
D2 (EDTA) ss	20 µl	D2 (EDTA) ss	14.2 µl
D1 (citrate) ds	20 µl	D1 (citrate) ds	19.7 µl
D2 (citrate) ds	20 µl	D2 (citrate) ds	21.8 µl
D1 (EDTA) ds	21 µl	D1 (EDTA) ds	25.1 µl
D2 (EDTA) ds	22 µl	D2 (EDTA) ds	26.2 µl
